# Supplementary material for: Cost-effectiveness analysis of percutaneous coronary intervention for single-vessel coronary artery disease: an economic evaluation of the ORBITA trial
Source: BMJ Open. 2021 Feb 9;11(2):e044054. doi: 10.1136/bmjopen-2020-044054 (PMC7875263; doi:10.1136/bmjopen-2020-044054)
Supplement: Supplementary data [file bmjopen-2020-044054supp001.pdf]

Parameters: ORBITA economic model

Parameter	Mean value	Probabilistic distribution	Source
**Cost of PCI**	1782	NA	HRG:EY41D, 2019/20 NHS National Tariff
**Cost of Cardiology outpatient visit**	78	NA	2019/20 NHS National Tariff
Weekly cost of pharmaceuticals			
**Aspirin**	0.13	NA	January 2019 NHS drug tariff
**Clopidogrel**	0.33	NA	January 2019 NHS drug tariff
**Statin**	0.24	NA	January 2019 NHS drug tariff
**ACE inhibitor**	0.53	NA	January 2019 NHS drug tariff
**Beta Blocker**	0.15	NA	January 2019 NHS drug tariff
**Calcium Channel Blocker**	0.16	NA	January 2019 NHS drug tariff
**Nitrate**	0.25	NA	January 2019 NHS drug tariff
**Nicorandil**	0.8	NA	January 2019 NHS drug tariff
**Ranolazine**	11.43	NA	January 2019 NHS drug tariff
**Total weekly cost without anti-angina drugs (PCI group scenario)**	1.11	NA	
Probability of taking drug type			
PCI group			
Aspirin	0.99	Beta	ORBITA Trial
Clopidogrel	1	Beta	ORBITA Trial
Statin	0.97	Beta	ORBITA Trial
ACE inhibitor	0.81	Beta	ORBITA Trial
Beta Blocker	0.81	Beta	ORBITA Trial
Calcium Channel Blocker	0.91	Beta	ORBITA Trial
Nitrate	0.66	Beta	ORBITA Trial
Nicorandil	0.48	Beta	ORBITA Trial
Ranolazine	0.07	Beta	ORBITA Trial
Placebo group			
Aspirin	0.97	Beta	ORBITA Trial
Clopidogrel	0.98	Beta	ORBITA Trial
Statin	0.96	Beta	ORBITA Trial
ACE inhibitor	0.79	Beta	ORBITA Trial
Beta Blocker	0.76	Beta	ORBITA Trial
Calcium Channel Blocker	0.89	Beta	ORBITA Trial
Nitrate	0.66	Beta	ORBITA Trial
Nicorandil	0.59	Beta	ORBITA Trial
Ranolazine	0.14	Beta	ORBITA Trial
Quality of life			
CAD (baseline)	0.77	Gamma	ORBITA Trial
Placebo	0.81		ORBITA Trial
PCI	0.83		ORBITA Trial
