# Supplementary material for: Cost-effectiveness analysis of percutaneous coronary intervention for single-vessel coronary artery disease: an economic evaluation of the ORBITA trial
Source: BMJ Open. 2021 Feb 9;11(2):e044054. doi: 10.1136/bmjopen-2020-044054 (PMC7875263; doi:10.1136/bmjopen-2020-044054)
Supplement: Author's manuscript [file bmjopen-2020-044054.draft_revisions.pdf]

BMJ Open is committed to open peer review. As part of this commitment we make the peer review history of every article we publish publicly available.

When an article is published we post the peer reviewers' comments and the authors' responses online. We also post the versions of the paper that were used during peer review. These are the versions that the peer review comments apply to.

The versions of the paper that follow are the versions that were submitted during the peer review process. They are not the versions of record or the final published versions. They should not be cited or distributed as the published version of this manuscript.

BMJ Open is an open access journal and the full, final, typeset and author-corrected version of record of the manuscript is available on our site with no access controls, subscription charges or pay-per-view fees (http://bmjopen.bmj.com).

If you have any questions on BMJ Open's open peer review process please email info.bmjopen@bmj.com

BMJ Open

Cost-effectiveness analysis of percutaneous coronary intervention for single vessel coronary artery disease: An economic evaluation of the ORBITA trial

Journal:	*BMJ Open*
Manuscript ID	bmjopen-2020-044054
Article Type:	Original research
Date Submitted by the Author:	07-Sep-2020

Complete List of Authors:	McCreanor, Victoria; Queensland University of Technology, AusHSI, Centre for Healthcare Transformation; Metro North Hospital and Health Service, Jamieson Trauma Institute, Royal Brisbane & Women's Hospital<br>Nowbar, Alexandra; Imperial College London, National Heart and Lung Institute; Imperial College Healthcare NHS Trust<br>Rajkumar, Christopher; Imperial College London, National Heart and Lung Institute; Imperial College Healthcare NHS Trust<br>Barnett, Adrian; Queensland University of Technology, AusHSI, Centre for Healthcare Transformation<br>Francis, Darrel; St Mary's Hospital, International Center for Cardiocirculatory Health<br>Graves, Nicholas; Duke-NUS Medical School, Health Services & Systems Research<br>Boden, William ; Boston University School of Medicine,<br>Weintraub, William; Georgetown University; MedStar Washington Hospital Center<br>Al-Lamee, Rasha; Imperial College London, National Heart and Lung Institute<br>Parsonage, William; Royal Brisbane and Women's Hospital, Cardiology; Queensland University of Technology, AusHSI, Centre for Healthcare Transformation

Keywords:	HEALTH ECONOMICS, Coronary intervention < CARDIOLOGY, Coronary heart disease < CARDIOLOGY

SCHOLARONE™
Manuscripts

I, the Submitting Author has the right to grant and does grant on behalf of all authors of the Work (as defined in the below author licence), an exclusive licence and/or a non-exclusive licence for contributions from authors who are: i) UK Crown employees; ii) where BMJ has agreed a CC-BY licence shall apply, and/or iii) in accordance with the terms applicable for US Federal Government officers or employees acting as part of their official duties; on a worldwide, perpetual, irrevocable, royalty-free basis to BMJ Publishing Group Ltd ("BMJ") its licensees and where the relevant Journal is co-owned by BMJ to the co-owners of the Journal, to publish the Work in this journal and any other BMJ products and to exploit all rights, as set out in our licence.

The Submitting Author accepts and understands that any supply made under these terms is made by BMJ to the Submitting Author unless you are acting as an employee on behalf of your employer or a postgraduate student of an affiliated institution which is paying any applicable article publishing charge ("APC") for Open Access articles. Where the Submitting Author wishes to make the Work available on an Open Access basis (and intends to pay the relevant APC), the terms of reuse of such Open Access shall be governed by a Creative Commons licence – details of these licences and which Creative Commons licence will apply to this Work are set out in our licence referred to above.

Other than as permitted in any relevant BMJ Author's Self Archiving Policies, I confirm this Work has not been accepted for publication elsewhere, is not being considered for publication elsewhere and does not duplicate material already published. I confirm all authors consent to publication of this Work and authorise the granting of this licence.

For peer review only

Cost-effectiveness analysis of percutaneous coronary intervention for single vessel coronary artery disease: An economic evaluation of the ORBITA trial

Authors: Victoria McCreanor[1], Alexandra Nowbar[3,4], Christopher Rajkumar[3,4], Adrian Barnett[1], Darrel P Francis[3,4], Nicholas Graves[1], William E Boden[5,6,7], William S Weintraub[8], Rasha Al-Lamee[3,4], William Parsonage[1,2]

Affiliations:

1. Australian Centre for Health Services Innovation (AusHSI), Institute of Health and Biomedical Innovation, Queensland University of Technology, Queensland, Australia
2. Royal Brisbane & Women's Hospital, Herston, Queensland, Australia
3. National Heart and Lung Institute, Imperial College London, London, UK
4. Imperial College Healthcare NHS Trust, London, UK
5. Boston University School of Medicine
6. Harvard Medical School
7. VA New England Healthcare System
8. MedStar Washington Hospital Center

Corresponding author contact details:

Prof. William Parsonage
Clinical Director, Australian Centre for Health Service Innovation, Queensland University of Technology
Senior Staff Cardiologist, Royal Brisbane & Women's Hospital
w.parsonage@mac.com

Word count: 4,031

For peer review only

ABSTRACT

Objective To evaluate the cost-effectiveness of percutaneous coronary intervention (PCI) compared to placebo in patients with single vessel coronary artery disease and angina despite anti-anginal therapy.

Design A cost-effectiveness analysis comparing PCI with placebo. A Markov model was used to measure incremental cost-effectiveness, in cost per Quality-Adjusted Life-Years (QALY) gained, over 12 months. Health utility weights were estimated using responses to the EuroQol 5 level questionnaire (EQ-5D-5L), from the ORBITA trial, and UK preference weights. Costs of procedures and follow-up consultations were derived from Healthcare Resource Group reference costs and drug costs from the NHS drug tariff. Probabilistic sensitivity analysis was undertaken to test the robustness of results to parameter uncertainty. Scenario analyses were performed to test the effect on results of reduced pharmaceutical costs in patients undergoing PCI, and the effect of patients crossing-over from placebo to PCI due to refractory angina within 12 months.

Setting Five UK NHS hospitals

Participants 200 adult patients with stable angina and angiographically severe single vessel coronary artery disease on anti-anginal therapy.

Interventions At recruitment patients received 6 weeks of optimisation of medical therapy for angina after which they were randomised to PCI or a placebo procedure.

Outcome measures Incremental cost-effectiveness ratio (ICER) expressed as cost (in £) per QALY gained for PCI compared to placebo.

Results The estimated ICER is £90,218/QALY gained when using PCI compared to placebo in patients receiving medical treatment for angina due to single vessel coronary artery disease. Results were robust under sensitivity analyses.

Conclusions The ICER for PCI compared to placebo, in patients with single vessel coronary artery disease and angina on anti-anginal medication, exceeds the threshold of £30,000 used by the National Institute of Health and Care Excellence when undertaking health technology assessment for the NHS context.

Trial registration London Central Research Ethics Committee (reference 13/LO/1340)

Summary of strengths and limitations:

- A strength of this research is that it is the first economic evaluation of PCI in patients with stable angina, using data from a randomised, placebo-controlled trial.
- This research is designed to provide useful and relevant information for decision-makers wanting to use cost-effectiveness evidence to make resource allocation decisions.
- A limitation of this study is that it uses data from only a short time horizon and extrapolation over a longer term may not be reliable.
- The research and results relate to only a subset of patients with stable coronary artery disease, and therefore may not be generalisable to a wider patient group.

For peer review only - http://bmjopen.bmj.com/site/about/guidelines.xhtml

BACKGROUND

Despite a substantial fall in age adjusted mortality rates, the prevalence of coronary heart disease has only decreased minimally over the last thirty years.[1] Coronary heart disease represents a major burden to the UK population with an estimated over 2 million people living with the disease and leading to approximately half a million inpatient episodes per year.[1] The cost of treating coronary heart disease in the UK is substantial. Between 1991 and 2014 prescriptions for all cardiovascular disease increased by 78% and although the number of coronary artery bypass operations has diminished since a peak in the 1990s the number of percutaneous coronary intervention (PCI) procedures has increased seven-fold over the same time.[2]

According to the National Reference Costs collection a total of 76,973 percutaneous transluminal coronary angioplasty procedures, Healthcare Resource Groups (HRGs) EY40 and EY41, were carried out by the National Health Service in 2017-18 and these 55,173 were coded as standard (non-complex) procedures at a total cost of £150,347,171.[3]

Published in 2017, the landmark Objective Randomised Blinded Investigation with optimal medical Therapy of Angioplasty in stable angina (ORBITA) study was the first trial to investigate the efficacy of PCI for symptom relief of stable angina in a double blind, placebo-controlled study. The trial randomised 200 patients with angina due to stable single vessel coronary heart disease to PCI or a placebo procedure with a primary end-point of exercise time at 6 weeks of follow up. The trial, which was more than adequately powered, showed that PCI when added to optimal medical therapy had no significant effect on the primary end-point.[4] Additionally, the study showed small, but not statistically significant, placebo-controlled differences in secondary endpoints of angina frequency and health related quality of life. Economic evaluation remains critically important in situations where clinical effectiveness of two interventions is similar but costs differ.

The ORBITA study remains the only blinded, randomised controlled trial of the efficacy of PCI in patients with angina and offers a unique opportunity to undertake an economic evaluation of this form of therapy.

The aim of this paper is to evaluate the cost-effectiveness of PCI compared to placebo when added to optimal medical therapy in patients with angina due to severe, single vessel coronary artery stenosis. Investing scarce resources for therapies that are not cost-effective reduces the aggregate of health in populations, as alternatives that deliver more health for the money are displaced.

METHODS

We conducted an economic evaluation, in the form of a cost-utility analysis, using data from the ORBITA trial, to assess the cost-effectiveness of PCI in patients with stable, single vessel coronary disease, in the context of the National Health Service of England.

Cost-utility analyses use health utility as the measure of health outcome. Health utility is a generic measure of a person's overall wellbeing, and takes a value between 1, full health, and 0, equivalent to being dead. It is measured using validated tools such as the EuroQol five-dimension quality of life instrument (EQ-5D)[5], and enables the calculation of Quality-Adjusted Life-Years (QALYs). QALYs are calculated by multiplying the health utility of a health state by the length of time a person

For peer review only - http://bmjopen.bmj.com/site/about/guidelines.xhtml

experiences that health state and are therefore superior to endpoints such as acute events or life expectancy, because they account for both length and quality of life. This is particularly important for chronic conditions, where the main treatment goal may be symptom relief.

We modelled costs and QALYs arising from the treatment effects of the ORBITA study, extrapolated to 12 months, and present the results as incremental cost-effectiveness ratios (ICERs) expressed as the cost per QALY gained.

Analyses were conducted using R statistical software (version 3.4.2) in the R Studio environment.[6, 7] Economic modelling was conducted using the *heemod* package in R.[8]

Model structure

For our analyses we used a Markov model. Markov models include health states, which patients transition through over time.[9] Patients have probabilities of moving during each cycle. Cycle length and the total number of cycles is determined by the disease and treatment trajectory. Our model uses weekly cycles, for 52 weeks. Each health state in a model has health outcomes and costs attached, and patients accrue these as they move through the model.

For chronic diseases, Markov models have advantages over other methods such as decision trees, as they enable patients to remain in one state over multiple cycles. Decision trees, by contrast, can become unwieldy because new branches may be needed for each chance of moving between health states.[9]

For our model of treatment for stable single vessel coronary artery disease, all patients enter the model with stable coronary disease and are treated with either medical therapy and placebo intervention, or medical therapy and percutaneous coronary intervention with stent implantation, Figure 1. The model uses data from the ORBITA trial, and models extrapolated costs and health outcomes to 12 months. It enables the comparison of costs and health outcomes for these patients under different treatment scenarios.

Model Assumptions

We did not include death or myocardial infarction in the model, because previous randomised trials comparing medical therapy and percutaneous coronary intervention have shown no difference in the risk of these events in patients with stable coronary artery disease. [10, 11]

We used a time frame of one year because, in previous open-label clinical trials comparing PCI to medical therapy for stable coronary artery disease, this is when a gain in quality of life from PCI is most pronounced. For example, in the COURAGE trial quality of life had diverged between the randomised groups after 4 weeks with the difference sustained at 12 months before converging and becoming not clinically significant at 24 months. [12]

Quality of life

We used the trial data for estimates of quality of life, based on all available measures of EQ-5D-5L at baseline and at completion of follow up at 6 weeks after randomisation according to the randomised allocation (intention to treat). These are shown as health utility weights derived from the EQ-5D-5L questionnaires using the value set for the UK population, derived by Devlin *et al*. [13] in the model.

For peer review only - http://bmjopen.bmj.com/site/about/guidelines.xhtml

In the ORBITA trial, the EQ-5D-5L questionnaire was administered to patients at three time points; enrolment, pre-randomisation and follow-up. This questionnaire has been validated for use as a health utility measure, for the purposes of economic evaluation.[5] The combination of responses to the five questions in the EQ-5D-5L are used to generate a health utility score between 0 and 1.

For the model, we used the mean health utility weight across all patients at enrolment for the CAD state and the mean health utility weight in each group, at completion of follow-up, for each of the treatment health states. We assumed that patients in these health states remained unchanged to 12 months after randomisation. The health utility weights used in the model are in Table 1.

Table 1: Health utility estimates from EQ-5D-5L data collected during the ORBITA trial. Higher scores indicate better health.

		**Utility weight**		
**Health state**	Number	Mean	SE	SD
**CAD (baseline)**	195	0.77	0.015	0.213
**Placebo**	91	0.81	0.021	0.221
**PCI**	104	0.83	0.023	0.212

CAD = coronary artery disease, Number = the number of patients in the sample, with a complete EQ-5D utility weight, used to estimate the mean and standard error. SE = standard error.

Costs of pharmaceutical therapy

We estimated mean weekly costs of pharmaceutical therapy in both placebo and percutaneous coronary intervention groups using the trial data, and the basic price from the NHS drug tariff. The supplementary materials to the ORBITA trial paper showed the number and percentage of people in each group taking each type of medication, and the details of the medical therapy protocol (See the ORBITA trial paper (supplementary materials of that paper, Table A3 in Appendix 4) [4]). We used those figures and the national tariffs for each drug, to calculate the mean costs in each group, as summarised in Table 2. The ORBITA medical therapy protocol is summarised in the supplementary materials, alongside the basic price from the January 2019 NHS drug tariff[14], as well as unit and weekly costs for each drug.

For peer review only - http://bmjopen.bmj.com/site/about/guidelines.xhtml

Table 2: Pharmaceutical costs for placebo and percutaneous coronary intervention groups using data from the ORBITA trial

Drug	ORBITA Protocol Dose	PCI Proportion taking	PCI Mean weekly cost	Placebo Proportion taking	Placebo Mean weekly cost
**Aspirin**	75mg OD	0.99	£0.13	0.97	£0.13
**Atorvastatin**	≥ 40mg OD	0.97	£0.23	0.96	£0.23
**Clopidogrel***	75mg OD	1.00	£0.33	0.98	£0.32
**Perindopril* (if known hypertension)**	≥ 4mg OD	0.81	£0.43	0.79	£0.42
**Bisoprolol***	≥ 5mg OD	0.81	£0.12	0.76	£0.11
**Amlodipine***	≥ 5mg OD	0.91	£0.15	0.89	£0.14
**Isosorbide mononitrate slow-release***	25mg OD	0.66	£0.16	0.66	£0.16
**Nicorandil**	10mg BD	0.48	£0.38	0.59	£0.47
**Ranolazine**	500mg BD	0.07	£0.76	0.14	£1.63
**Mean weekly total cost**			**£2.69**		**£3.62**

*BD = twice daily, OD = once daily, * = or equivalent*

We used average weekly costs of medical therapy in the percutaneous coronary intervention group of £2.69 per week, and £3.62 per week in the placebo group (Table 2).

For the scenario analysis where patients undergoing PCI no longer require anti-anginal medication, we used an average weekly medical therapy cost of £1.11, calculated by removing costs for anti-anginal medications in the PCI group, in Table 2.

Cost of percutaneous coronary intervention

The national tariff sets out the prices and payment rules used by NHS providers and commissioners of care, to deliver cost-effective care.[15] We used the 2019/20 Healthcare Resource Group (HRG) reference costs for Standard Percutaneous Transluminal Coronary Angioplasty (code EY41D), £1,782.[16, 17] That group includes PCI, with insertion of one or two drug-eluting stents, in patients with up to three comorbidities, such as diabetes and hypertension.

Cost of cardiology clinic visits

We included costs of the ongoing visits to cardiology clinics. We estimated that those undergoing PCI would attend once, three months after their procedure, and those who were treated with placebo would attend at three, six and nine months. These visits were costed according to the 2019/20 NHS National Tariff for outpatient cardiology attendances by a single professional at £78 per patient per visit.[16, 17]

Model outcomes

When the Markov model is run, the costs and health outcomes arising from the patient's transition through the health states are summed to estimate the total costs and health outcomes for each

For peer review only - http://bmjopen.bmj.com/site/about/guidelines.xhtml

treatment; optimal medical therapy plus PCI or optimal medical therapy plus placebo. Results are presented as an incremental cost-effectiveness ratio (ICER). ICERs are simple ratios dividing the change in costs and the change in health outcomes resulting from an investment in a new services or health technology, in this case the use of percutaneous coronary intervention. ICERs show the additional costs required to achieve one additional unit of health benefit, one QALY, and are expressed as the cost per QALY gained.

ICERs are assessed against a threshold for cost-effectiveness. In the United Kingdom, the National Institute for Health and Care Excellence (NICE) currently uses a threshold of £20,000 to £30,000 per QALY gained, with an accepted upper limit of £30,000, which we used for our analyses.[18]

It is usual to discount future costs and health outcomes when running health economic models.[19] Future costs and health outcomes were discounted at a rate of 3.5% per annum, as recommended by NICE. [20]

Probabilistic sensitivity analysis

To test the sensitivity of the model to uncertainty in parameter estimates, we conducted probabilistic sensitivity analyses.[9] For each parameter where there was uncertainty in the mean, we created a distribution around our baseline estimate.

We modelled uncertainty in the costs of pharmaceuticals by varying the percentages of people prescribed each drug type, using beta distributions. We did not vary the dosages as we felt this might create unrealistic combinations of prescriptions and dosages which would not reflect reality. Conventionally, gamma distributions are used for healthcare costs, due to their skewed shape which allows for a small number of patients to incur very high costs. However, we did not feel that a gamma distribution would be appropriate for pharmaceutical costs, because there is likely to be little variation across patients. NHS costs for pharmaceuticals are low and most people follow similar treatment regimes.

We used a normal distribution to model uncertainty in the estimates of health utility, using the mean and standard error from the EQ-5D-5L questionnaire (See Table 1).

For the costs of the PCI procedure, we used only the lowest bracket of HRG EY41. This relates to the least complex patients with the fewest comorbidities. The ORBITA patients were not complex, due to design and inclusion criteria of the study, so we did not consider it appropriate to model higher procedure costs in this analysis. Similarly, we did not model any uncertainty in the cost of cardiology outpatient visits.

We took 5,000 random samples from the distributions for each relevant parameter, generating 5,000 ICERS. We calculated the probability of cost-effectiveness by calculating the proportion of the simulated ICERs that fall below the cost-effectiveness threshold of £30,000 per QALY gained.

Scenario analyses

PCI for refractory angina in control patients

We tested the effect on the economic outcome of a scenario of patients in the control group returning with refractory symptoms requiring PCI. We modelled increasing proportions of control

For peer review only - http://bmjopen.bmj.com/site/about/guidelines.xhtml

patients returning for PCI within 12 months in increments of 20% and recalculated the ICER for comparison to the base case analysis that assumed no crossover.

Reduced pharmaceutical cost of treating angina in patient following PCI

We also tested the effect on the economic outcome of a scenario where patients treated with PCI would require less anti-anginal therapy than control patients. We repeated the base-case model removing all costs for anti-anginal drugs (but not costs of antiplatelet and lipid lowering drugs) in the patients undergoing PCI from the time of the procedure until 12 months.

Public and Public Involvement

How was the development of the research question and outcome measures informed by patients' priorities, experience, and preferences? This paper is a secondary analysis of the ORBITA study. The original ORBITA study was designed in close cooperation with patients and the public. The Patient and Public Coordinator at the NIHR Imperial Biomedical Research Unit and the Research Design Service were engaged in reviewing the trial protocol and patient information documents including the patient information leaflet and consent form.

How did you involve patients in the design of this study? The ORBITA Focus Group, composed of patients who participated in ORBITA, have provided input and support with secondary analyses.

Were patients involved in the recruitment to and conduct of the study? No. There was public and patient involvement in aspects of planning the ORBITA trial, however patients were not involved in recruitment or running of the study.

How will the results be disseminated to study participants? Results will be fed back to the ORBITA focus group and disseminated through formal publications.

For randomised controlled trials, was the burden of the intervention assessed by patients themselves? The burden of the intervention was not specifically assessed by patients. However, the ORBITA trial, patient information and consent forms were designed in collaboration with Patient and Public Coordinators, and all patients gave informed consent to participate.

For peer review only

RESULTS

Baseline model outcomes

The baseline cost-effectiveness results are in Table 3. The results show an increase in costs for the PCI group compared with the placebo group, which is accompanied by only a very small health gain of 18 QALYs per 1,000 patients. The estimated cost-effectiveness ratio is £90,218 per QALY gained when using PCI compared to placebo in addition to medical therapy, in this group of patients. This is far higher than the £30,000 threshold used by NICE, and therefore, in these patients, use of PCI would not be considered cost-effective.

For peer review only - http://bmjopen.bmj.com/site/about/guidelines.xhtml

Table 3: Cost-effectiveness results for a cohort of 1,000 patients

Treatment	Total Costs	Total QALYs	Cost Difference	QALY difference	ICER
Placebo	£410,405	796.092			
PCI	£1,995,418	813.661	£1,585,012	17.569	£90,218*

ICER calculated prior to rounding.

Scenario analyses: varying the percentage of placebo group patients returning for PCI

The results for this scenario analysis, where an increasing proportion of patients in the placebo group go on to receive PCI within the year, are shown in Table 4. The results show that the incremental cost-effectiveness ratio remains above £30,000 per QALY gained when even 80% of patients return to undergo PCI within the first year following initiation of anti-anginal therapy.

Table 4: Cost-effectiveness results for a cohort of 1,000 patients, where the percentage of placebo patients returning for PCI within one year is varied

Scenario: percent crossing over from Placebo	Total Costs	Total QALYs	Cost Difference†	QALY difference†	ICER*
20%	£740,538	797.87	£1,254,880	15.791	£79,469
40%	£1,070,460	799.815	£924,958	13.846	£66,804
60%	£1,399,549	802.023	£595,869	11.637	£51,203
80%	£1,725,707	804.751	£269,711	8.91	£30,271

†compared with the PCI group. *ICERs calculated prior to rounding

ICER calculated prior to rounding.

Scenario analysis: lower medical therapy costs following PCI

The results for the scenario analysis where those undergoing PCI are able to stop all anti-anginal medications are in Table 5. In this scenario the incremental cost-effectiveness ratio remains high, at £85,576 per QALY gained.

For peer review only - http://bmjopen.bmj.com/site/about/guidelines.xhtml

For peer review only

Table 5: Cost-effectiveness results for a cohort of 1,000 patients, where those undergoing PCI have stopped all anti-anginal medical therapy.

Treatment	Total Costs	Total QALYs	Cost Difference	QALY difference	ICER
**Placebo**	£410,405	796.092			
**PCI**	£1,913,852	813.661	£1,503,447	17.569	£85,576*

*ICER calculated prior to rounding.

Results of the Probabilistic sensitivity analysis

The results of the probabilistic sensitivity analysis are shown graphically in Figure 2, and summarised in Table 6.

In Figure 2 there is only one point for the placebo group, because this is the comparison group. It is clear from the plot that fewer blue points fall under the £30,000 threshold for cost-effectiveness than fall above it. This indicates that PCI is unlikely to be cost-effective at that threshold, compared with the placebo in patients on anti-anginal therapy.

Table 6 confirms these results showing that PCI was cost-effective compared to placebo in only 11% of simulations. There is a low probability of PCI being cost-effective in this single vessel coronary artery disease patient group.

Table 6: Proportion of simulations where each treatment strategy is cost-effective

Treatment group	Cost-effective (% of simulations)
**Placebo**	89
**PCI**	11

DISCUSSION

This study describes the cost-effectiveness of PCI compared to placebo when added to optimal medical therapy, using data derived from the only double blind, randomised trial of PCI in patients with stable single vessel coronary artery disease.

There are three important findings. Firstly, the baseline analysis shows, with a high level of certainty, that PCI for angina relief, in patients with single vessel coronary disease on anti-anginals, requires a cost per extra QALY that exceeds thresholds typically used for cost-effectiveness in the NHS. Secondly, even if PCI eliminated the need for anti-anginal therapy this has minimal effect on cost-effectiveness. Finally, even if placebo patients were to present with symptoms requiring PCI further down the line, it would require this to happen in more than 80% of patients for the placebo arm to become less cost effective than the PCI arm. These results appear to be driven by the relatively small difference in quality of life improvements in the PCI group, compared with placebo.

Our baseline analysis generated an ICER of £90,218 per QALY gained when comparing PCI to placebo and this exceeds the threshold of £30,000 often used by NICE when considering the cost-effectiveness of treatments. Supporting the baseline estimate, the probabilistic analysis demonstrates a very high level of certainty in the model outcomes, with less than 12% of simulations

For peer review only - http://bmjopen.bmj.com/site/about/guidelines.xhtml

favouring routine use of PCI in this patient group. There has been vigorous debate about this threshold recently. Research from the Centre for Health Economics at the University of York, focussing on opportunity cost, or what is foregone when investment in a new technology or service displaces current services, suggests that the threshold should be about £13,000 per QALY gained [21-23]. This means that investments in new services with ICERs above £13,000 per QALY gained would result in overall harm to NHS patients, as resources would be drawn away from services which would generate more QALYs for the same investment.

These findings support, on a cost-effectiveness basis, the strategy of anti-anginal medication as first line, as advised by international guidelines.[24, 25] In clinical practice, non-PCI patients might need additional visits to maintain anti-anginal therapy levels similar to ORBITA. However, even when notional costs of such additional visits are added, the magnitude of the difference between the ICER and the cost-effectiveness threshold suggests that the non-PCI approach remains economically advantageous.

The ORBITA study protocol set out to continue medical therapy unchanged until completion of clinical follow-up at 6 weeks following randomisation. To account for the possibility that patients treated with PCI would require less anti-anginal therapy over a longer horizon we repeated the analysis with a scenario where the costs of all anti-anginal drugs were withdrawn in the PCI group but continued in the placebo group. Because drugs used to treat angina are relatively cheap this had a minimal effect on the ICER for PCI, which was reduced to £85,576 per QALY.

Another important consideration is that the relatively short 6-week clinical follow-up of the ORBITA study may have masked longer term clinical benefits of PCI over medical therapy. One specific concern is that, over a longer horizon, patients may experience more angina symptoms than detected at 6 weeks and that the placebo effect may attenuate over time. To allow for this in our economic evaluation, we explored the proportion of patients that would need to return requiring PCI for refractory symptoms of angina within 12 months, despite optimal medical therapy, before it would be cost-effective to provide routine PCI in all patients. We found that more than 80% of patients would need to return for PCI within 12 months, before it became cost-effective to provide PCI to all patients, at the outset. This rate of crossover seems unlikely based on experience from previous randomised comparisons of PCI and medical therapy for stable coronary artery disease. For example, in the COURAGE trial over 4.6 years of follow-up additional revascularisation occurred in 32.6% of medically treated patients compared to 21.1% of those randomised to PCI. [11] In the ORBITA trial itself, all patients had already been referred for clinical PCI, and therefore after completing their participation in the study, it was assumed that all placebo patients would then go forward for clinical PCI, and indeed most did so. This was not driven by the results of the trial because the results were not available at the time. In light of the lack of significant differences in the primary and secondary endpoints of the study related to angina; functional capacity, frequency of angina and quality of life at 6 weeks, there might be less bias towards PCI as a default treatment.[4]

Limitations

The ORBITA trial included only patients with stable single vessel coronary artery disease, and therefore we cannot generalise the results to patients with more complex disease. It is possible that patients with multi-vessel disease, more symptoms, or a higher ischaemic burden may have more to

For peer review only - http://bmjopen.bmj.com/site/about/guidelines.xhtml

gain from PCI. The ORBITA 2 trial is currently underway, and is designed to investigate the placebo-controlled efficacy of PCI in a wider clinical population.[26]

We did not model variation in costs for PCI procedures, because we felt that this best reflected the nature of the procedures and patients included in the study. However, because we used the lowest relevant HRG tariff, this would bias the model in favour of PCI. Inclusion of higher costs would have shown PCI to be less cost-effective.

We assumed that health states remained stable from the 6 weeks follow-up to a horizon of 12 months, for the purpose of the analysis. Given that there was no difference between the groups for the key clinical endpoints in ORBITA at 6 weeks, we assumed that the effect of the intervention on quality of life was sustained over 12 months. Other factors that would affect quality of life over the longer horizon (for example other ill health) are likely to be randomly distributed between the groups and unlikely to have biased our findings.

Our model was run for a 12-month period and did not include events such as death or myocardial infarction. As noted in the methods, we did not model these events because earlier trials have demonstrated no difference, in patients with stable disease, for the two treatments examined here.[10, 11]

Similarly, we did not run the model over a longer horizon, because other research has shown that improvements in symptom relief and quality of life in this patient group are most pronounced in the short term. [12] We acknowledge, however, that the outcome of the model would be sensitive to more sustained improvements in symptoms and quality of life, even if these effects are relatively small in magnitude. Publication of the outcome of studies with longer term follow up (such as the recently published ISCHEMIA trial) may help to inform models with a longer horizon. However, the open-label design of these trials leaves measures of health related quality of life susceptible to bias that can only be controlled for in double blind, placebo controlled studies, of which ORBITA remains the only trial of this kind at this point in time.

We were only able to partially allow for the possible effects of withdrawal of anti-anginal therapy in patients following PCI. For example, we are unable to allow for a negative effect of continuing medical therapy (medication disutility), which is likely to be greater than zero.[27] The disutility attributable to continuation of anti-anginal medication is unknown but is likely to be a complex net effect of beneficial and adverse effects. Given that patients are advised to continue with other medications (including lipid lowering and antiplatelet agents) the effect of any disutility of continued anti-anginal therapy on our conclusions is likely to be negligible. Additionally, we have observed that in 'real-world' practice anti-anginal drugs are often continued in patients following PCI, so our scenario analysis of withdrawal of all anti-anginal therapy in patients following PCI is also likely to be biased in favour of PCI.[28]

Our analysis is based specifically on costs relating to NHS England and cannot therefore be directly translated to other health systems. However, the costs of PCI are relatively low in the publicly-funded NHS by comparison to privately-funded healthcare systems. Our model is readily able to be adapted to accommodate costs incurred in different health systems.

For peer review only - http://bmjopen.bmj.com/site/about/guidelines.xhtml

Conclusions

Our results show that for patients with stable single vessel coronary artery disease and angina on medical therapy, there is a low probability that it is cost effective to add PCI even in a healthcare system where PCI is relatively inexpensive. This conclusion is resistant to the possibility that PCI may lead to a reduction in down-stream costs for anti-anginal drugs and cardiology outpatient visits and/or an increase in subsequent PCI procedures for refractory symptoms.

FIGURE LEGEND

Figure 1: Markov model of health states

CAD = Coronary Artery Disease, PCI = Percutaneous Coronary Intervention

Figure 2: Scatter plot showing results of the probabilistic sensitivity analysis for a cohort of 1000 patients.

OMT = Optimal Medical Therapy, PCI = Percutaneous Coronary Intervention, Thr = Threshold, QALY = Quality-Adjusted Life-Year

SOURCES OF FUNDING

Dr Al-Lamee, Professor Francis, Dr Nowbar and Dr Rajkumar acknowledge support from the NIHR Imperial Biomedical Research Centre (P74227).

ETHICS

This work is included under the terms of the original ethical approval for the ORBITA study obtained from the Central London Ethics Committee (REC reference 13/LO/1340).

ACKNOWLEDGEMENTS

Computational resources and services used in this work were provided by the eResearch Office, Queensland University of Technology, Brisbane, Australia.

The ORBITA Focus Group is thanked for its ongoing contribution to the development and dissemination of secondary analyses and subsequent research following the original ORBITA study.

CONFLICTS OF INTEREST

RAL declares speakers' fees from Philips Volcano, Menarini Pharmaceuticals, outside this work. No other disclosures.

For peer review only - http://bmjopen.bmj.com/site/about/guidelines.xhtml

For peer review only

AUTHOR CONTRIBUTIONS

WP and VM planned the concept and design of the study. VM undertook the modelling and data analyses. WP drafted the initial manuscript. All authors (AB, AN, CR, DF, NG, RA, VM, WB, WP and WW) reviewed the initial manuscript and provided feedback on the design and interpretation of the results. All authors (AB, AN, CR, DF, NG, RA, VM, WB, WP and WW) contributed to intellectual content and critical revisions the work. All authors (AB, AN, CR, DF, NG, RA, VM, WB, WP and WW) give final approval of the version to be published and agree to be accountable for all aspects of the work.

DATA SHARING STATEMENT

The authors commit to making the relevant anonymised patient level data available on reasonable request.

For peer review only

REFERENCES

1. Heart & Circulatory Disease Statistics 2019. 2019 [Accessed on 13/06/2019
2. Bhatnagar P, Wickramasinghe K, Wilkins E, et al. Trends in the epidemiology of cardiovascular disease in the UK. *Heart*. 2016;102(24):1945-52.
3. Reference costs. 2018 [Available from: https://improvement.nhs.uk/resources/reference-costs. Accessed on 03/04/2019
4. Al-Lamee R, Thompson D, Dehbi H-M, et al. Percutaneous coronary intervention in stable angina (ORBITA): a double-blind, randomised controlled trial. *Lancet*. 2017.
5. EuroQol Research Foundation. EQ-5D. Rotterdam.
6. R Core Team. R: A Language and Environment for Statistical Computing. Vienna, Austria: R Foundation for Statistical Computing; 2017.
7. RStudio Team. RStudio: Integrated Development Environment for R. Boston, MA: RStudio, Inc.; 2016.
8. Filipović-Pierucci A, Zarca K, Wiener M, et al. heemod: Markov Models for Health Economic Evaluations. 2015.
9. Briggs A, Sculpher MJ, Claxton K. Chapter 2: Key aspects of decison modelling for economic evaluation. Decision Modelling for Health Care Economic Evaluation. Oxford: Oxford University Press; 2006.
10. De Bruyne B, Pijls NH, Kalesan B, et al. Fractional flow reserve-guided PCI versus medical therapy in stable coronary disease. *N Engl J Med*. 2012;367(11):991-1001.
11. Boden WE, O'Rourke RA, Teo KK, et al. Optimal medical therapy with or without PCI for stable coronary disease. *N Engl J Med*. 2007;356(15):1503-16.
12. Weintraub WS, Spertus JA, Kolm P, et al. Effect of PCI on quality of life in patients with stable coronary disease. *N Engl J Med*. 2008;359(7):677-87.
13. Devlin NJ, Shah KK, Feng Y, et al. Valuing health-related quality of life: An EQ-5D-5L value set for England. *Health Econ*. 2018;27(1):7-22.
14. Drug Tariff Part VIII. 2019 [Available from: https://www.nhsbsa.nhs.uk/pharmacies-gp-practices-and-appliance-contractors/drug-tariff/drug-tariff-part-viii. Accessed on 25/02/2019
15. National tariff payment system. 2019 [Available from: https://improvement.nhs.uk/resources/national-tariff/. Accessed on 04/04/2019
16. NHS Improvement. 2019/20 National Tariff Payment System: national prices and prices for emergency care services. 2019.
17. NHS Improvement. 2019/20 National Tariff Payment System. 2019.
18. McCabe C, Claxton K, Culyer AJ. The NICE cost-effectiveness threshold: what it is and what that means. *Pharmacoeconomics*. 2008;26(9):733-44.
19. Gray A, Clarke P, Wolstenholme J, et al. Applied Methods of Cost-effecitvness Analysis in Health Care. Gray A, Briggs A, editors. Oxford: Oxford University Press; 2011.
20. York Health Economics Consortium. Discount Rate. York: 2016 [Available from: https://yhec.co.uk/glossary/discount-rate/. Accessed on 10/12/2019 2019]
21. Approval of new drugs by NICE is doing more harm than good. The NHS is paying too much for new drugs [Press Release]. 2015.
22. Claxton K, Martin S, Soares M, et al. Methods for the Estimation of the NICE Cost Effectiveness Threshold. York: Centre for Health Economics, The University of York 2013.
23. University of York. Health Opportunity Costs (Estimating health opportunity costs in the NHS and other health care systems) Methods for estimation of the NICE cost-effectiveness threshold. York: 2015 [Available from: https://www.york.ac.uk/che/research/teehta/thresholds/. Accessed on 25/02/2019
24. Fihn SD, Gardin JM, Abrams J, et al. 2012 ACCF/AHA/ACP/AATS/PCNA/SCAI/STS Guideline for the Diagnosis and Management of Patients With Stable Ischemic Heart Disease A Report of the American College of Cardiology Foundation/American Heart Association Task Force on Practice Guidelines, and the American College of Physicians, American Association for Thoracic Surgery,

For peer review only - http://bmjopen.bmj.com/site/about/guidelines.xhtml

Preventive Cardiovascular Nurses Association, Society for Cardiovascular Angiography and Interventions, and Society of Thoracic Surgeons. *J Am Coll Cardiol*. 2012;60(24):e44-e164.
25. Montalescot G, Sechtem U, Achenbach S, et al. 2013 ESC guidelines on the management of stable coronary artery disease: the Task Force on the management of stable coronary artery disease of the European Society of Cardiology. *Eur Heart J*. 2013;34(38):2949-3003.
26. ClinicalTrials.gov. A Placebo-controlled Trial of Percutaneous Coronary Intervention for the Relief of Stable Angina (ORBITA-2). USA: 2018 [Available from: https://clinicaltrials.gov/ct2/show/NCT03742050. Accessed on 30/04/2019
27. Fontana M, Asaria P, Moraldo M, et al. Patient-Accessible Tool for Shared Decision Making in Cardiovascular Primary Prevention: Balancing Longevity Benefits Against Medication Disutility. *Circulation*. 2014;129(24):2539-46.
28. McCreanor V. Identifying high-value care for coronary artery disease in Australia. Brisbane: Queensland University of Technology; 2019.

For peer review only

For peer review only - http://bmjopen.bmj.com/site/about/guidelines.xhtml

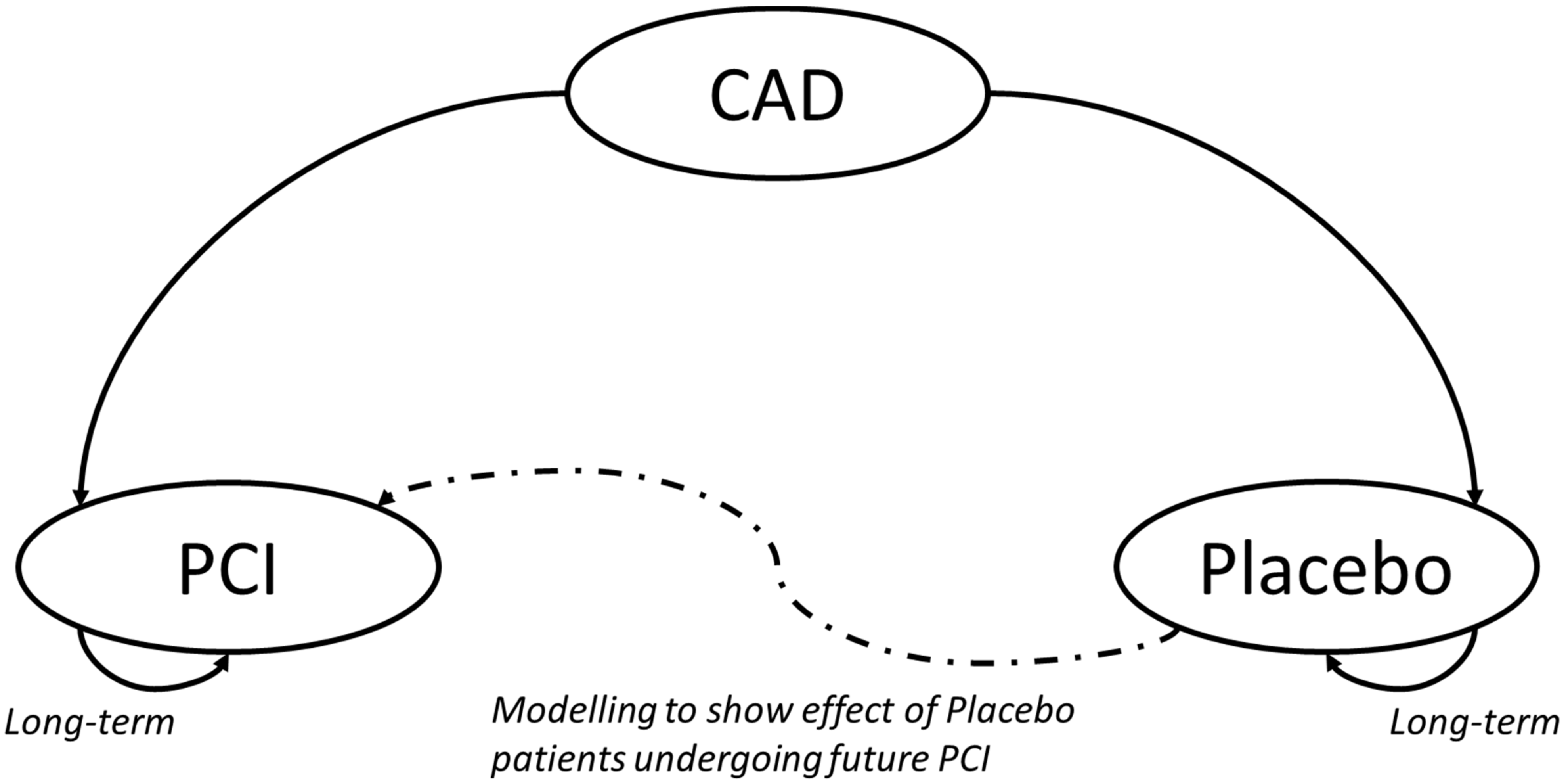
CAD
PCI
Placebo
Long-term
Modelling to show effect of Placebo patients undergoing future PCI
Long-term

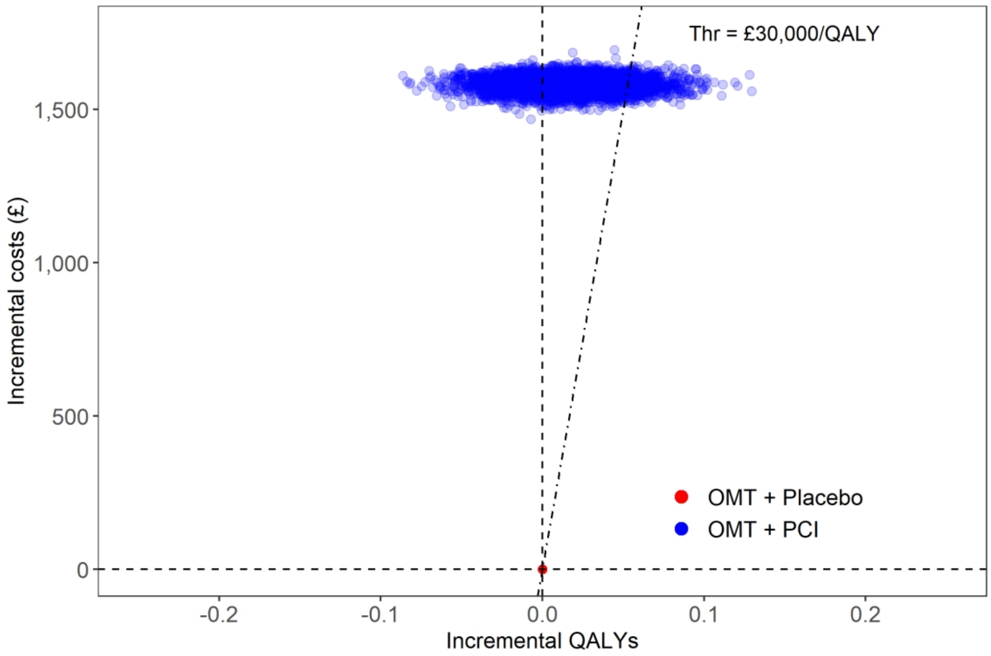

Scatter plot showing results of the probabilistic sensitivity analysis for a cohort of 1,000 patients.

OMT = Optimal Medical Therapy, PCI = Percutaneous Coronary Intervention, Thr = Threshold, QALY = Quality-Adjusted Life-Year

190x127mm (300 x 300 DPI)

Parameters: ORBITA economic model

Parameter	Mean value	Probabilistic distribution	Source
**Cost of PCI**	1782	NA	HRG:EY41D, 2019/20 NHS National Tariff
**Cost of Cardiology outpatient visit**	78	NA	2019/20 NHS National Tariff
Weekly cost of pharmaceuticals			
**Aspirin**	0.13	NA	January 2019 NHS drug tariff
**Clopidogrel**	0.33	NA	January 2019 NHS drug tariff
**Statin**	0.24	NA	January 2019 NHS drug tariff
**ACE inhibitor**	0.53	NA	January 2019 NHS drug tariff
**Beta Blocker**	0.15	NA	January 2019 NHS drug tariff
**Calcium Channel Blocker**	0.16	NA	January 2019 NHS drug tariff
**Nitrate**	0.25	NA	January 2019 NHS drug tariff
**Nicorandil**	0.8	NA	January 2019 NHS drug tariff
**Ranolazine**	11.43	NA	January 2019 NHS drug tariff
**Total weekly cost without anti-angina drugs (PCI group scenario)**	1.11	NA	
Probability of taking drug type			
PCI group			
Aspirin	0.99	Beta	ORBITA Trial
Clopidogrel	1	Beta	ORBITA Trial
Statin	0.97	Beta	ORBITA Trial
ACE inhibitor	0.81	Beta	ORBITA Trial
Beta Blocker	0.81	Beta	ORBITA Trial
Calcium Channel Blocker	0.91	Beta	ORBITA Trial
Nitrate	0.66	Beta	ORBITA Trial
Nicorandil	0.48	Beta	ORBITA Trial
Ranolazine	0.07	Beta	ORBITA Trial
Placebo group			
Aspirin	0.97	Beta	ORBITA Trial
Clopidogrel	0.98	Beta	ORBITA Trial
Statin	0.96	Beta	ORBITA Trial
ACE inhibitor	0.79	Beta	ORBITA Trial
Beta Blocker	0.76	Beta	ORBITA Trial
Calcium Channel Blocker	0.89	Beta	ORBITA Trial
Nitrate	0.66	Beta	ORBITA Trial
Nicorandil	0.59	Beta	ORBITA Trial
Ranolazine	0.14	Beta	ORBITA Trial
Quality of life			
CAD (baseline)	0.77	Gamma	ORBITA Trial
Placebo	0.81		ORBITA Trial
PCI	0.83		ORBITA Trial

For peer review only

CHEERS Checklist

Section	Item No	Recommendation	Reported on page No
Title and Abstract			
Title	1	Identify the study as an economic evaluation or use more specific terms such as cost-effectiveness analysis and describe the interventions compared	1
Abstract	2	Provide a structured summary of objectives, perspective, setting, methods (including study design and inputs), results (including base case and uncertainty analyses) and conclusions	2
Introduction			
Background and objectives	3	Provide an explicit statement of the broader context for the study. Present the study question and its relevance for health policy or practice decisions	3
Methods			
Target population and subgroups	4	Describe characteristics of the base case population and subgroups analysed, including why they were chosen	3
Setting and location	5	State relevant aspects of the system(s) in which the decision(s) need(s) to be made	3
Study perspective	6	Describe the perspective of the study and relate this to the costs being evaluated	3
Comparators	7	Describe the interventions or strategies being compared and state why they were chosen	3
Time horizon	8	State the time horizon(s) over which costs and consequences are being evaluated and say why appropriate	4
Discount rate	9	Report the choice of discount rate(s) used for costs and outcomes and say why appropriate	7
Choice of health outcomes	10	Describe what outcomes were used as the measure(s) of benefit in the evaluation and their relevance for the type of analysis performed	3,4-5
Measurement of effectiveness	11a	*Single study-based estimates*: describe the design feature of the single effectiveness study and why the single study was a sufficient source of clinical effectiveness data	5

For peer review only

Section	Item No	Recommendation	Reported on page No
	11b	*Synthesis-based estimates*: describe fully the methods used for identification of included studies and synthesis of clinical effectiveness data	N/A
Measurement and valuation of preference based outcomes	12	If applicable, describe the population and methods used to elicit preferences for outcomes	5
Estimating resources and costs	13a	*Single study-based economic evaluation*: Describe approaches used to estimate resource use associated with the alternative interventions. Describe primary or secondary research methods for valuing each resource item in terms of its unit cost. Describe any adjustments made to approximate opportunity costs.	4-5
	13b	*Model-based economic evaluation*: Describe approaches and data sources used to estimate resource use associated with model health states. Describe primary or secondary research methods for valuing each resource item in terms of its unit cost. Describe any adjustments made to approximate opportunity costs.	N/A
Currency, price date, and conversion	14	Report the dates of the estimated resource quantities and unit costs. Describe methods for adjusting estimated unit costs to the year of reported costs if necessary. Describe methods for converting costs into a common currency base and the exchange rate.	6, 7
Choice of model	15	Describe and give reasons for the specific type of decision-analytic model used. Providing a figure to show model structure is strongly recommended	4-5
Assumptions	16	Describe all structural or other assumptions underpinning the decision-analytical model	5-7
Analytical methods	17	Describe all analytical methods supporting the evaluation. This could include methods for dealing with skewed, missing, or censored data; extrapolation methods; methods for pooling data; approaches to validate or make adjustments (such as half cycle corrections) to a model; and methods for handling population heterogeneity and uncertainty	5-7

For peer review only

Section	Item No	Recommendation	Reported on page No
Results			
Study parameters	18	Report the values, ranges, references and, if used, probability distributions for all parameters. Report reasons or sources for distributions used to represent uncertainty where appropriate. Providing a table to show the input values is strongly recommended.	5-7, Suppl. Materials
Incremental costs and outcomes	19	For each intervention, report mean values for the main categories of estimated costs and outcomes of interest, as well as mean differences between the comparator groups. If applicable, report incremental cost-effectiveness ratios.	8
Characterising uncertainty	20a	*Single study-based economic evaluation*: Describe the effects of sampling uncertainty for the estimated incremental cost and incremental effectiveness parameters, together with the impact of methodological assumptions (such as discount rate, study perspective).	9-10
	20b	*Model-based economic evaluation*: Describe the effects on the results of uncertainty for all input parameters, and uncertainty related to the structure of the model and assumptions.	N/A
Characterising heterogeneity	21	If applicable, report differences in costs, outcomes, or cost-effectiveness that can be explained by variations between subgroups of patients with different baseline characteristics or other observed variability in effects that are not reducible by more information.	N/A
Discussion			
Study findings, limitations, generalisability, and current knowledge	22	Summarise key study findings and describe how they support the conclusions reached. Discuss limitations and the generalisability of the findings and how the findings fit with current knowledge.	10-12
Other			
Source of funding	23	Describe how the study was funded and the role of the funder in the identification, design, conduct and reporting of the analysis. Describe other non-monetary sources of support.	12

For peer review only

Section	Item No	Recommendation	Reported on page No
Conflicts of interest	24	Describe any potential for conflict of interest of study contributors in accordance with journal policy. In the absence of a journal policy, we recommend authors comply with International Committee of Medical Journal Editors recommendations.	13

For peer review only
